# Periconceptional Maternal Folic Acid Use of 400 µg per Day Is Related to Increased Methylation of the *IGF2* Gene in the Very Young Child

**DOI:** 10.1371/journal.pone.0007845

**Published:** 2009-11-16

**Authors:** Régine P. Steegers-Theunissen, Sylvia A. Obermann-Borst, Dennis Kremer, Jan Lindemans, Cissy Siebel, Eric A. Steegers, P. Eline Slagboom, Bastiaan T. Heijmans

**Affiliations:** 1 Obstetrics and Gynecology/Division of Obstetrics and Prenatal Medicine, Erasmus MC, University Medical Center, Rotterdam, The Netherlands; 2 Epidemiology, Erasmus MC, University Medical Center, Rotterdam, The Netherlands; 3 Clinical Genetics, Erasmus MC, University Medical Center, Rotterdam, The Netherlands; 4 Pediatrics, Erasmus MC, University Medical Center, Rotterdam, The Netherlands; 5 Clinical Chemistry, Erasmus MC, University Medical Center, Rotterdam, The Netherlands; 6 Molecular Epidemiology, Leiden University Medical Center, Leiden, The Netherlands; 7 Child Health Care Center CAREYN, Rotterdam, Netherlands; National Institute of Child Health and Human Development/National Institutes of Health, United States of America

## Abstract

**Background:**

Countries worldwide recommend women planning pregnancy to use daily 400 µg of synthetic folic acid in the periconceptional period to prevent birth defects in children. The underlying mechanisms of this preventive effect are not clear, however, epigenetic modulation of growth processes by folic acid is hypothesized. Here, we investigated whether periconceptional maternal folic acid use and markers of global DNA methylation potential (S-adenosylmethionine and S-adenosylhomocysteine blood levels) in mothers and children affect methylation of the insulin-like growth factor 2 gene differentially methylation region (*IGF2* DMR) in the child. Moreover, we tested whether the methylation of the *IGF2* DMR was independently associated with birth weight.

**Methodology/Principal Findings:**

*IGF2* DMR methylation in 120 children aged 17 months (SD 0.3) of whom 86 mothers had used and 34 had not used folic acid periconceptionally were studied. Methylation was measured of 5 CpG dinucleotides covering the DMR using a mass spectrometry-based method. Children of mother who used folic acid had a 4.5% higher methylation of the *IGF2* DMR than children who were not exposed to folic acid (49.5% vs. 47.4%; p = 0.014). *IGF2* DMR methylation of the children also was associated with the S-adenosylmethionine blood level of the mother but not of the child (+1.7% methylation per SD S-adenosylmethionine; p = 0.037). Finally, we observed an inverse independent association between *IGF2* DMR methylation and birth weight (−1.7% methylation per SD birthweight; p = 0.034).

**Conclusions:**

Periconceptional folic acid use is associated with epigenetic changes in *IGF2* in the child that may affect intrauterine programming of growth and development with consequences for health and disease throughout life. These results indicate plasticity of *IGF2* methylation by periconceptional folic acid use.

## Introduction

Every year around 8 million children are born with a serious birth defect worldwide. Folate deficiency during conception up to the third month of gestation, i.e., periconceptional period, is an etiological factor in several birth defects. Randomized controlled trials have shown that periconceptional synthetic folic acid use prevents neural tube defects [1]. For that reason periconceptional folic acid in a dose of 400 µg per day has been promoted to all women planning pregnancy [2].

Over the last decade, several campaigns were started to improve the awareness of the importance of periconceptional use of synthetic folic acid in tablets. Furthermore, folic acid fortification of food has been introduced in the US, Canada and Chile [3]. Since the implementation of these measures, a significant decrease in birth rates of neural tube defects, orofacial clefts, congenital heart defects and diaphragmatic hernia has been reported [4], [5], [6], [7]. However, periconceptional folic acid use has also been reported to have adverse effects including an elevated risk of pyloric stenosis, obstructive urinary tract defects, obesity, insulin resistance and colon cancer [7], [8], [9].

The mechanisms underlying the beneficial and adverse effects of periconceptional folic acid use are largely unclear. Following current thinking about the developmental origins of health and disease, an altered epigenetic regulation of growth processes induced by periconceptional folic acid may contribute to both the immediate effects and chronic disease associations in later life [10], [11]. Epigenetic regulation determines the potential of a genomic region to become transcribed [12]. The best understood epigenetic mechanism is the methylation of cytosine-guanine (CpG) dinucleotides in the DNA of mammals. Methyl donors, including folic acid, are required to establish and maintain DNA methylation. Methyl groups for DNA methylation reactions are supplied by demethylating the activated form of methionine into S-adenosylmethionine (SAM), to form S-adenosylhomocysteine (SAH) and homocysteine. In agreement with their crucial role in methylation reactions, in human SAM and SAH plasma levels and the SAM/SAH ratio are frequently used markers of global DNA methylation potential [13], [14].

Direct evidence that the availability of methyl donors during gestation is required to establish and maintain DNA methylation patterns comes from experiments in the yellow *A^vy^ agouti* mice [15]. Supplementing the diet of pregnant dams with methyl donors, including folic acid, results in silencing of the *agouti* gene due to DNA methylation resulting in offspring with a mainly brown coat colour and a lower tendency for obesity, cancer and diabetes. We recently observed that similar mechanisms may play a role in humans. Periconceptional exposure to famine during the Dutch Famine at the end of WWII was associated with a persistently lower methylation of the maternally imprinted insulin-like growth factor 2 (*IGF2*) gene [16]. *IGF2* is an embryonic growth factor that is expressed in most tissues and regulated in rats by periconceptional nutrient intake [17]. Complete loss of methylation at the *IGF2* differentially methylated region (DMR) results in biallelic expression of *IGF2* and is associated with an increased risk of colorectal adenoma [18]. *IGF2* imprinting defects also underlie Beckwith-Wiedemann syndrome which is characterized by overgrowth [19]. Here, we hypothesize that periconceptional folic acid use by the mother may have consequences for *IGF2* DMR methylation of the child with a subsequent effect on intrauterine growth as reflected in birth weight.

## Results

The quantitative traits, including birth weight and the biochemical markers of global DNA methylation and folate, were similar according to periconceptional folic acid use for both mothers and children (table 1). The relative methylation of the *IGF2* DMR was 4.5% higher in folic acid exposed children as compared with non exposed children (absolute methylation 0.495 (SE 0.004) vs. 0.474 (0.007); p = 0.014, table 2. In the linear mixed model analysis additionally adjustment for maternal education level revealed an adjusted p-value of 0.009, table 3. Higher levels of methylation were also observed for individual CpG dinucleotides comprising the *IGF2* DMR, particularly for CpG #4, although not always significantly.

**Table 1:** Quantitative traits according to maternal periconceptional folic acid use.

	Mothers			Children		
	No Folic Acid (n = 34)	Yes Folic Acid (n = 86)	*P-*value	No Folic Acid (n = 34)	Yes Folic Acid (n = 86)	*P-*value
**Male, n (%)**	-	-		20 (59%)	50 (58%)	0.945
**Age, years**	32.6 (0.8)	32.2 (0.4)	0.624	-	-	-
**Age, months**	-	-	-	17.1 (0.5)	17.3 (0.2)	0.709
**Birth weight, grams**	-	-	-	3363 (98)	3490 (64)	0.287
**Gestational age, weeks**	-	-	-	39.4 (0.3)	39.7 (0.2)	0.496
**Biochemistry**						
**SAM, µmol/L**	79.9 (2.5)	80.2 (1.3)	0.897	102.7(3.3)	106.4 (2.1)	0.363
**SAH, µmol/L**	15.0 (0.6)	14.5 (0.3)	0.438	18.5 (1.0)	17.3 (0.5)	0.278
**SAM/SAH**	5.5 (0.2)	5.7 (0.1)	0.357	6.1 (0.4)	6.6 (0.2)	0.267
**Folate, nmol/L**						
**serum**	15.3 (0.9)	17.8 (1.2)	0.189	31.5 (2.5)	32.1 (1.6)	0.748
**red blood**	687 (70)	720 (30)	0.589	973 (72)	1064 (41)	0.245

Data are presented in numbers (percentages) and mean (standard error).

Abbreviations: SAM, S-adenosylmethionine; SAH, S-adenosylhomocysteine.

**Table 2:** *IGF2* DMR methylation in the child according to maternal periconceptional folic acid use of 400 µg per day.

	No Folic Acid (n = 34)	Yes Folic Acid (n = 86)	*P*-value
**Complete DMR**	0.474 (0.007)	0.495 (0.004)	0.014
**CpG #1**	0.473 (0.009)	0.484 (0.005)	0.292
**CpG #2&3**	0.334 (0.006)	0.348 (0.004)	0.059
**CpG #4**	0.590 (0.016)	0.632 (0.010)	0.023
**CpG #5**	0.511 (0.011)	0.516 (0.080)	0.602

Linear Mixed Model analysis. Independent absolute methylation of the CpG dinucleotides without adjustments is presented in mean and (standard error).

**Table 3:** *IGF2* DMR methylation in the child and independent factors of the mother and the child.

Factors	Mother	*P*-value	Child	*P*-value
**Folic acid use**	+4.5% (1.8)	0.014	-	-
**Female sex**	-	-	+2.0% (1.6)	0.232
**Age**	−0.4% (0.8)	0.585	−0.7% (1.0)	0.478
**Birth weight**	-	-	−1.7% (0.8)	0.034
**Gestational age**	-	-	−0.9% (0.8)	0.276
**Biochemistry**				
**SAM, µmol/L**	+1.7% (0.8)	0.037	+1.2% (0.8)	0.129
**SAH, µmol/L**	+0.8% (0.8)	0.331	+0.1% (0.8)	0.882
**SAM/SAH**	+0.0% (0.8)	0.985	+0.3% (0.8)	0.717

Linear Mixed Model analysis. Data are presented in percentage (standard error) of mean change in relative methylation. For independent quantitative parameters the change in relative methylation is given per SD-change in that parameter. The p-value of the significant association of periconceptional folic acid use and *IGF2* DMR methylation was additionally adjusted for maternal education. The p-value for the significant association between maternal SAM and IGF2 DMR methylation was also adjusted for maternal education and the SAM concentration of the child. The p-value for the significant association between IGF2 DMR methylation and birth weight was additionally adjusted for periconceptional folic acid use and gestational age at delivery.

Next, we tested the association of other variables in mother and child with *IGF2* DMR methylation in children (table 3). In addition to periconceptional maternal folic acid use, a higher maternal SAM concentration, but not that of the child, was associated with a higher *IGF2* methylation in the child (p = 0.037). This association remained significant after additional adjustment for maternal education and the SAM concentration of the child (p_adjusted_ = 0.047).

To test for a possible phenotypic consequence of changes in *IGF2* methylation, we analyzed the relationship with birth weight. A 1.7% higher *IGF2* methylation in the child was associated with one SD decrease in birth weight of 584 grams (p = 0.034), which was independent of periconceptional exposure to folic acid and gestational age at delivery (p_adjusted_ = 0.041).

## Discussion

The key finding of our study is that periconceptional folic acid use of the mother is related to an increased methylation of the *IGF2* DMR of the child. The reported stability of *IGF2* DMR methylation up to middle age [20], [21] supports the interpretation that the *IGF2* methylation changes we observed are explained by periconceptional folic acid exposure. The difference in DNA methylation associated with folic acid exposure is remarkably similar to our previous observation of a 5.2% reduced *IGF2* methylation after periconceptional exposure to famine [18]. The opposite direction of the associations suggests that the availability of methyl donors during the periconceptional period may affect *IGF2* DMR methylation. We further hypothesized that changes in *IGF2* DMR methylation would influence intrauterine growth. Our study indeed indicated an association between *IGF2* DMR methylation and birth weight as surrogate for intrauterine growth, but not between periconceptional folic acid use and birth weight.

Compared to our findings in humans, the size of the effects on DNA methylation of prenatal exposures have found to be comparable in sheep but are larger in rodents [22], [23], [24]. In this comparison we have to emphasize that human populations are inevitably more heterogeneous than the inbred rodents kept at the same, well-controlled environmental conditions. The different effects in animal studies can also be due to the common use of a combined intervention consisting of multiple methyl donors and/or protein deficiency instead of folic acid only. Lastly, the larger effects in rodents are shown in other tissues than in blood, which are not readily accessible from human study subjects.

In addition, our study indicated an association between higher *IGF2* methylation and lower birth weight. This inverse association is compatible with a relative intrauterine silencing of the embryonic growth factor *IGF2* resulting in reduced growth [25], [26]. This links our data to the finding that *IGF2* loss of imprinting leads to somatic overgrowth (Beckwith-Wiedemann syndrome) [27] and possibly colorectal cancer [20] although we cannot exclude the explanation that the change in *IGF2* methylation marks possible greater changes elsewhere in the genome that underlie the association observed. Periconceptional folic acid use may contribute to the restoration of a loss of imprinting. It remains to be established whether DNA methylation changes contribute to the adverse effects reported for periconceptional folic acid use [7], [8], [9], [28]. Studies are required to establish optimal timing, dose and type (natural folate or synthetic folic acid) to prevent birth defects and at the same time minimize adverse effects later in life.

From this study reveals that periconceptional folic acid use is associated with epigenetic changes in IGF2. However, in contrast with large mother-child cohorts, we did not find a positive association between periconceptional folic acid use and higher birth weight [29], [30]. It is known that women using periconceptional folic acid supplements are generally more health conscious and higher educated. Furthermore, the women exposed to the Dutch famine were not only deprived of folate, but also of other essential macro- and micronutrients that serve as methyldonors, e.g., methionine. Thus, it should be emphasized that many factors together including other genes besides periconceptional folic acid contribute to birth weight. This may explain the absent association between periconceptional folic acid use and birth weight in our study.

Periconceptional use of folic acid did not affect average levels of the biomarkers SAM, SAH or SAM/SAH measured at 17 months in the mother and in the child. However, we found a significant correlation between the maternal SAM concentration at the study moment and *IGF2* methylation of the child. The developmental hypothesis of health and disease states that periconceptional exposures may affect metabolic imprinting of the child. This is in line with our finding that periconceptional folic acid use can affect the metabolic imprinting of the methylation pathway of the child. Of note, this association was not influenced by fortification of food with folic acid which is absent in The Netherlands, UK and other European countries, which may have strengthened the observed associations.

Although we did not measure levels of biomarkers of methylation in the periconception period, their levels will have been comparable to those we measured 17 months after delivery. This is substantiated by Nurk et al. showing that the biomarkers of methylation show a limited variability in the periconception period and over a subsequent period of 1–2 years [31]. Furthermore, there are no substantial differences in preconceptional maternal dietary habits and lifestyles and those 1 to 1.5 year post partum which affect these biomarkers [32].

A limitation of both our study and others is that they relied on genomic DNA extracted from whole blood so that heterogeneity in cell populations may have contributed to the outcomes [18]. Our study likely is less sensitive to such heterogeneity because the epigenetic state of imprinted loci is less dependent on cell differentiation and, importantly, a previous study showed that when demethylation of *IGF2* DMR was observed in peripheral blood lymphocytes of an individual, this was also found in colon tissue [20], which has a distinct embryologic origin (endoderm and mesoderm, respectively). The finding that the common epigenetic variation in *IGF2* DMR might influence birth weight is intriguing but should be interpreted with care because the sample size was relatively small and other (non)genetic factors are also involved. It is currently unknown whether modestly increased *IGF2* DMR methylation upregulates *IGF2* expression. Furthermore, it has not been established whether such quantitative differences measured in lymphocytes mark a soma-wide phenomenon as was suggested for loss-of-imprinting of the *IGF2* DMR [20]. Therefore, in future human studies the sampling of different tissues should be performed.

Our study provides the first evidence that periconceptional folic acid use may be related to DNA methylation in the child. Moreover, such DNA methylation changes may have phenotypic consequences as illustrated by the association between higher *IGF2* methylation and decreased birth weight. A simple preventive strategy as periconceptional folic acid use may affect epigenetic control and as such may link the prevention of intrauterine development, i.e., birth defects such as neural tube defects, and growth due to a loss of imprinting with the risk of chronic diseases in these children throughout life. It has to be established how folic acid intake affects the epigenetic regulation of sets of relevant genes and whether adverse effects are to be expected from an altered methylation at such loci. Given the ongoing exposure it is timely to monitor the (long term) effect on DNA methylation also of other lifestyles and environmental influences, such as overnutrition, fortification of food, smoking, stress and the use of assisted reproductive techniques.

## Methods

### Ethics statements

The study protocol was approved by the Central Committee for Human Research (CCMO) in The Hague, The Netherlands, and the Medical Ethical and Institutional Review Board of the Erasmus MC, University Medical Center in Rotterdam, The Netherlands. All mothers gave their written informed consent and mothers and their partner on behalf of their participating child.

### Study design and population

In a cross-sectional study mothers and children between 12 and 18 months of age were enrolled via public health centers in Rotterdam, the Netherlands between October 2003 and January 2007. The Dutch health care system includes a standardized and regular check up of all newborns for health, growth and development by physicians trained in child health care. Children were eligible as controls if they did not have a major congenital malformation or chromosomal defect according to the medical records from the regular check up at the child health center. These mothers and their healthy nonmalformed child served as controls in the previously described HAVEN-study [33], [34]. Mothers and children were studied at the standardized study moment of around 17 months after delivery of the index child, at which both blood samples for DNA methylation and folate in serum and red blood cells of the child and questionnaire data via the mother on periconceptional exposures, such as folic acid use, were obtained. For 186 mother-child pairs biomarkers of global methylation and blood samples for DNA methylation and folate in serum and red blood cells were available. Because we aimed to show an effect of periconceptional folic acid use, in particular an extreme effect, the 48 mothers who used partially folic acid during this period were excluded. 40 Mothers had completely refrained from periconception folic acid use and 98 reported the use of folic acid according to the Dutch recommendation of a daily intake of a folic acid containing preparation of 400 µg from at least 4 weeks before until 8 weeks after conception. Six mother-child pairs of whom the mother had not used folic acid were excluded because insufficient genomic DNA was available of the child for bisulfite treatment resulting in 34 unexposed mother-children pairs eligible for the current epigenetic study. For technical reasons 86 mother-child pairs were randomly selected from the exposed group, so that the total number of mothers and children studies was 120.

The questionnaires providing information on general traits, folic acid use and birth weight filled out by the mother at home were checked for completeness and consistency at the hospital visit during the standardized study moment by the researcher. We extracted data on maternal age and folic acid use, and age, gender, birth weight and gestational age at delivery of the child.

The standardization of the blood sampling, plasma handling, extraction of genomic DNA and measurement of SAM, SAH and folate were described previously and are reported as control data in the comparison of cases with a congenital heart defect [33], [34].

### DNA Methylation

DNA methylation measurements were performed on genomic DNA extracted from whole blood samples obtained from the children. Bisulphite treatment was carried out on 0.5 µg genomic DNA using the EZ 96-DNA methylation kit (Zymo Research). The 120 samples were blinded as to exposure status and split into two equal groups with a similar distribution in exposure status thus preventing possible batch effects. Subsequently, to assess *IGF2* DMR methylation 5 CpG dinucleotides of the *IGF2* DMR (chr11:2,126,035-2,126,372 in NCBI build 36.1) was measured in triplicate using a mass spectrometry-based method (Epityper, Sequenom)[20]. The quantitative accuracy and concordance with clonal polymerase chain reaction bisulphite sequencing is well-established [21]. Two CpG dinucleotides confounded by SNPs were discarded so that the current study reports on CpG dinucleotides located at positions 41 (CpG #1),57 and 60 (#2&3; adjacent CpGs that could not be resolved individually, 202 (#4) and 251 (#5) bp in the amplicon targeting the *IGF2* DMR.

### Statistical analyses

Differences in quantitative traits in mothers and in children according to maternal periconception folic acid use were tested using an independent t-test. Differences in gender distribution were tested using a chi-square test. *IGF2* DMR methylation was assessed by measuring multiple CpG sites that are correlated [21]. Raw methylation data can still be used (instead of for example averaging over the various CpG dinucleotides that differ in DNA methylation level) by applying linear mixed models. Linear mixed models may be viewed as an extension of a t-test that accounts for the correlation between methylation of CpG dinucleotides and methylation data missing at random [16], [18]. When applied to the analysis of single CpG dinucleotides without adjustment for covariates, a linear mixed model and a t-test yield identical results. For the analysis of the complete *IGF2* DMR, methylation data all CpG dinucleotides were entered in the model with the study subject identifier as a random effect to account for the correlation between CpG dinucleotides. The CpG dinucleotide identifier, the exposure status and other traits (e.g., SAM concentration or birth weight) or covariates (e.g., bisulfite plate) were entered as fixed effects. When testing single CpG dinucleotides, the study subject and CpG nucleotide identifiers were removed from the linear mixed model. Before testing independent associations of quantitative traits with *IGF2* DMR methylation, Z-scores were calculated so that the resulting estimated effect size indicates the methylation change per standard deviation (SD) change in the parameter tested. Z-score does not affect a variable otherwise than standardising the mean to 0 and the SD to 1 and assists in interpreting the results.

The p-value of the significant association of periconceptional folic acid use and *IGF2* DMR methylation was additionally adjusted for maternal education. The p-value for the significant association between maternal SAM and IGF2 DMR methylation was also adjusted for maternal education and the SAM concentration of the child. The p-value for the significant association between IGF2 DMR methylation and birth weight was additionally adjusted for periconceptional folic acid use and gestational age at delivery. All p-vales were two-sided and all statistical analyses were performed using SPSS 16.0.
